# Supplementary material for: Identification of photoperiod-induced specific miRNAs in the adrenal glands of Sunite sheep (Ovis aries)
Source: Front Vet Sci. 2022 Jul 22;9:888207. doi: 10.3389/fvets.2022.888207 (PMC9354845; doi:10.3389/fvets.2022.888207)
Supplement: Supplementary file 1 [file Data_Sheet_1.ZIP › Supplementary file/miRDeep2 file.zip!/miRDeep2 file/Know_Structure/LP42A3/oar-mir-106b.pdf]

miRBase precursor : oar-mir-106b
Total read count : 4355
oar-miR-106b read count : 1616
remaining reads : 2739

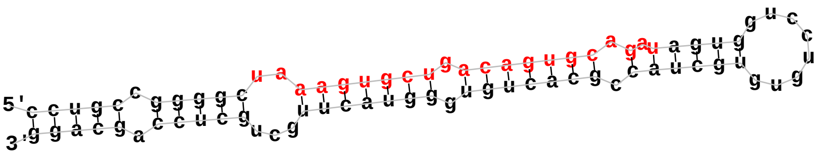

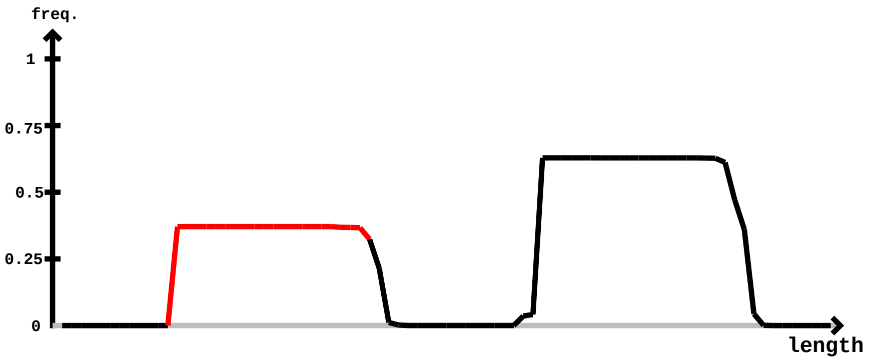


```
             oar-miR-106b
5'- ccugccggggcuaaagugcugacagugcagauagugguccugugugcuaccgcacuguggguacuugcugcuccagcagg   -3'    exp
    (((((.(((((..(((((((.(((((((.(.(((((........)))))).))))))).)))))))...))))).)))))    reads      mm         sample
    ...........uaaagugcugacagugc....................................................    12         0          seq
    ...........uaaagugcugacagugca...................................................    2          0          seq
    ...........uaaagugcugacagugcag..................................................    4          0          seq
    ...........uaaagugcugacagugcaga.................................................    190        0          seq
    ...........uaaagugcugacagugcagau................................................    472        0          seq
    ...........uaaagugcugacagugcagaua...............................................    884        0          seq
    ...........uaaagugcugacagugcagauag..............................................    43         0          seq
    ...........uaaagugcugacagugcagauagu.............................................    8          0          seq
    ............aaagugcugacagugcagaua...............................................    1          0          seq
    ..................................ugguccugugugcuaccgcac.........................    1          0          seq
    ...............................................uaccgcacuguggguacuu..............    3          0          seq
    ...............................................uaccgcacuguggguacuugc............    13         0          seq
    ...............................................uaccgcacuguggguacuugcu...........    138        0          seq
    ...............................................uaccgcacuguggguacuugcugc.........    1          0          seq
    ................................................accgcacuguggguacuugc............    1          0          seq
    ................................................accgcacuguggguacuugcu...........    14         0          seq
    ................................................accgcacuguggguacuugcug..........    6          0          seq
    ................................................accgcacuguggguacuugcugc.........    4          0          seq
    .................................................ccgcacuguggguacuug.............    3          0          seq
    .................................................ccgcacuguggguacuugc............    55         0          seq
    .................................................ccgcacuguggguacuugcu...........    457        0          seq
    .................................................ccgcacuguggguacuugcug..........    476        0          seq
    .................................................ccgcacuguggguacuugcugc.........    1375       0          seq
    .................................................ccgcacuguggguacuugcugcu........    187        0          seq
    .................................................ccgcacuguggguacuugcugcuc.......    4          0          seq
    ..................................................cgcacuguggguacuugcugc.........    1          0          seq
```
